# Focused very high-energy electron beams as a novel radiotherapy modality for producing high-dose volumetric elements

**DOI:** 10.1038/s41598-019-46630-w

**Published:** 2019-07-25

**Authors:** K. Kokurewicz, E. Brunetti, G. H. Welsh, S. M. Wiggins, M. Boyd, A. Sorensen, A. J. Chalmers, G. Schettino, A. Subiel, C. DesRosiers, D. A. Jaroszynski

**Affiliations:** 1 SUPA, Department of Physics, University of Strathclyde, G4 0NG Glasgow, UK; 2 Strathclyde Institute of Pharmacy and Biomedical Sciences, University of Strathclyde, G4 0RE Glasgow, UK; 3 Wolfson Wohl Cancer Research Centre, Institute of Cancer Sciences, Glasgow University, G61 1QH Glasgow, UK; 4 University of Glasgow, G12 8QQ Glasgow, UK; 5 National Physical Laboratory, Hampton Road, TW11 0LW Teddington, Middlesex UK; 6 Indiana University, School of Medicine, Department of Radiation Oncology, RT 041 Indianapolis, USA; 7 University of Surrey, Department of Physics, Guilford, GU2 7XH UK

**Keywords:** Cancer imaging, Radiotherapy

## Abstract

The increased inertia of very high-energy electrons (VHEEs) due to relativistic effects reduces scattering and enables irradiation of deep-seated tumours. However, entrance and exit doses are high for collimated or diverging beams. Here, we perform a study based on Monte Carlo simulations of focused VHEE beams in a water phantom, showing that dose can be concentrated into a small, well-defined *volumetric element*, which can be shaped or scanned to treat deep-seated tumours. The dose to surrounding tissue is distributed over a larger volume, which reduces peak surface and exit doses for a single beam by more than one order of magnitude compared with a collimated beam.

## Introduction

The main goal of radiotherapy is to kill cancer cells whilst minimising damage to healthy tissue. Photons in the megavoltage energy range, and electrons up to 25 MeV, have been commonly used as external beam therapies since the 1950s^1^. Initially, the delivery of radiation was not sufficiently precise to achieve tumoricidal doses without causing collateral damage to healthy tissue^2,3^. Since then, advances in radiation delivery techniques, including 3-Dimensional Conformal Radiotherapy (3DCRT)^4^, Intensity Modulated Radiation Therapy (IMRT)^5^, Volumetric Arc Therapy (VMAT)^6^, and Stereotactic Radiosurgery (SRS, e.g. with Gamma Knife^R^)^7^, have improved dose conformity to the target volume. Hadron therapy improves conformity further because dose is concentrated within the Bragg peak^8^. However, the high cost and large size of hadron accelerators and their associated beam transport systems^9^ limit their availability. These factors are stimulating the search for less expensive but precise radiotherapy solutions.

**Figure 1:**
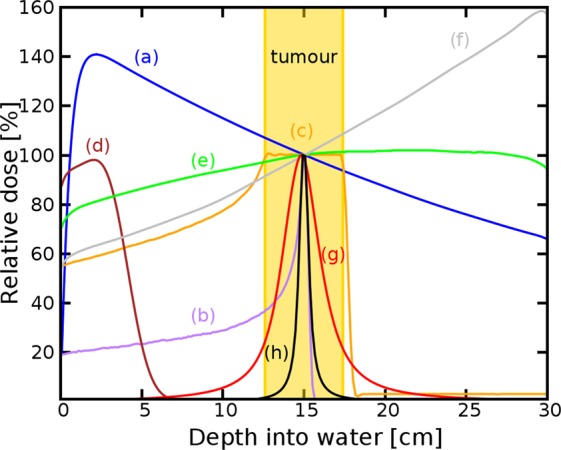
Percentage on-axis depth-dose (PDD) curves of different types of radiation in a water phantom. (**a**) 6 MV Photons, (**b**) Bragg peak 147 MeV protons, (**c**) spread-out Bragg peak, (**d**) 10 MeV electrons, (**e**) collimated 200 MeV electrons, (**f**) collimated 2 GeV electrons, (**g**) 200 MeV electrons focused at 15 cm, (**h**) 2 GeV electrons focused at 15 cm. For comparison, each curve is normalised to the dose at the reference depth (15 cm) apart from the 10 MeV electron beam, which is normalised to its peak dose. Curves (**a–f**) correspond to a Gaussian beam with full-width-at-half-maximum (FWHM) diameter of 15.9 cm, matching the size of the focused beams (curves **g,h**) at the phantom entrance.

Targeting of deep-seated tumours requires accurate delivery of high radiation doses through thick layers of tissue. Conventional MV clinical photons deposit their maximum dose within the first 5 cm of tissue, as shown in Fig. 1, followed by exponentially diminishing dose deposition (curve **a**). In comparison, mono-energetic protons deposit their maximum dose in the Bragg peak (curve **b**), have a much lower entrance dose and exhibit rapid falloff of dose near the end of their range. The entire tumour volume can be covered by superimposing multiple beams with different energies to produce a spread-out-Bragg peak region (curve **c**). However, this results in an increased entrance dose. Here we investigate an alternative modality based on focused Very High Energy Electron (VHEE) beams.

**Figure 2:**
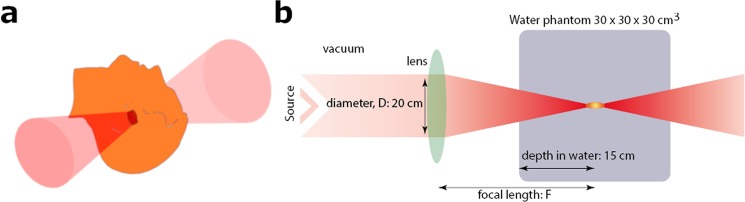
Focused VHEE therapy. (**a**) focused VHEE beam. Schematic diagram **(b**) showing an electron beam focused into a water phantom by an ideal magnetic lens. The beam diameter at the lens is fixed at 20 cm. Different focusing configurations are studied by varying the position and focal length of the lens, while keeping the focus position fixed, at the reference depth (15 cm). The lens and beam positions can also be changed to scan the single volumetric element over an arbitrary volume.

Monte Carlo simulations show that electrons with energies greater than 50 MeV (VHEEs) can reach deep-seated tumours^10–13^; in contrast to current 4–25 MeV clinical electron beams^14^ (curve **d**). Further theoretical studies indicate that VHEEs can enhance the sparing of critical structures, while providing similar, and sometimes better, target coverage^10,13,15–18^ than photons and protons, and reduced susceptibility to tissue inhomogeneities^11,18,19^. However, a collimated VHEE beam creates a nearly uniform dose distribution, as shown in Fig. 1, curve **e**, which results in high entrance and exit doses. At GeV energies (curve **f**), electrons deposit a lower entrance dose, but the dose increases continuously as they propagate, which results in a higher exit dose. To overcome these adverse depth-dose profiles we investigated the properties of focused VHEE beams, as shown schematically in Fig. 2. Preliminary modelling data indicate that focused 200 MeV and 2 GeV beams achieve highly localised dose deposition at the target depth (15 cm), as shown by curves **g** and **h** in Fig. 1. 200 MeV electrons are within the established energy range of VHEE beams^12^, while 2 GeV electrons, here referred to as ultra-high electron energy (UHEE), penetrate more deeply into material and exhibit further reductions in scattering^20^. The aim of this study is to investigate the feasibility of treating deep-seated tumours using VHEEs, and to validate our main hypothesis that high-energy focused beams can precisely concentrate radiation dose into a small well-defined volumetric element deep inside the body, while sparing adjacent tissue, as schematically depicted in Fig. 2. To do this we have used a general purpose Monte Carlo numerical code (FLUKA^21^) to model the propagation of VHEE beams in a water phantom for different focusing strengths. Previous experiments^22^ and Monte Carlo simulations^23^ with electron energies up to 70 MeV have shown that strong magnetic fields inside the phantom can reduce lateral scattering and concentrate the dose deposition. The use of magnetic fields outside the phantom has also been reported for low energy electrons^24^ and weakly focused VHEEs^25^. In this study we consider high electron energies and a wide range of focusing strengths. We have simulated a beam with an initial diameter *D*, focused to a point at the centre of a 30 × 30 × 30 cm^3^ water phantom^26^ (used as tissue equivalent material), by an ideal magnetic lens with a focal length *F*. A practical implementation of this focusing system could be realised using large-aperture electromagnetic quadrupoles arranged in a FODO lattice (Focus-Drift-Defocus-Drift). We show that dose in the phantom is concentrated into a small volume with a size that depends on the focusing geometry, and is characterised by the *f-number*, which is defined as *f* = *F*/*D* (as in optics). Strongly focused beams are consistent with *f-numbers* less than 3.8.

## Results

Monte Carlo simulations, as described in Methods, were used to model dose deposition by VHEE and UHEE beams in water for different *f-numbers*. We estimated on-axis, off-axis, surface and exit doses, and the contributions of secondary particles, such as positrons, neutrons and muons. We also investigated the activity induced in a phantom containing bone and muscle material.

**Figure 3:**
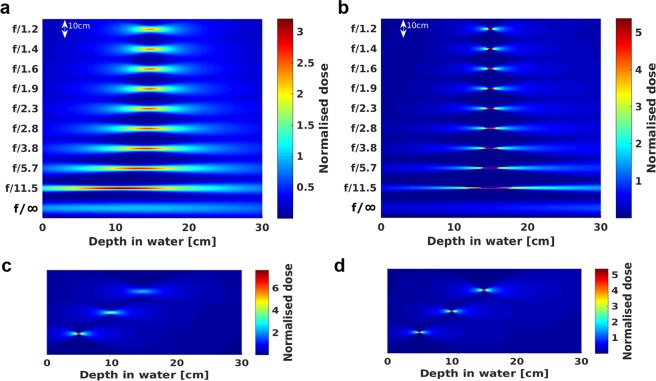
2D depth-dose deposition map in the water phantom. (**a)** 200 MeV and (**b)** 2 GeV electron beams focused 15 cm from the entrance of the water phantom for *f/11.5*–*f/1.2* and collimated geometry (*f/*∞); (**c**) 200 MeV and (**d**) 2 GeV electron beams focused at 5, 10 and 15 cm from the entrance of the water phantom for *f/1.2*. The dose is normalised to the maximum dose of a collimated beam.

Figure 3 shows 2D depth-dose distributions of 200 MeV (**a**) and 2 GeV (**b**) electron beams focused at 15 cm depth with *f-numbers* between *f/1.2* and *f/11.5*, and a reference collimated beam (*f*/∞). Electrons initially propagate in a vacuum and the beam diameter (FWHM) at the entrance of the phantom varied between 2.5 cm (for *f/*∞) and 15.9 cm (for *f/1.2*). We also ran simulations where the electrons propagate for 9.1–214 cm in air, corresponding to focal length of the strongest (*f/1.2*) and weakest (*f/11.5*) focusing, and observed the beam size to increase by less than 1% at the entrance of the phantom. Scattering in water has more prominent effect on the beam distribution. It shifted the focus towards the phantom entrance by approximately 0.4–4.5 cm for 200 MeV, and 0.1–0.3 cm for 2 GeV, for the chosen *f-numbers*, with a small increase in transverse size. Focusing at depths of 5, 10 and 15 cm was also investigated by varying the source to surface distance to displace the position of the high-dose *volumetric element*, as shown in Fig. 3c,d for 200 MeV and 2 GeV electron beams and *f/1.2*. Peak dose significantly decreased with depth for the 200 MeV energy beam, whereas little change was observed for the more penetrating 2 GeV beam.

**Figure 4:**
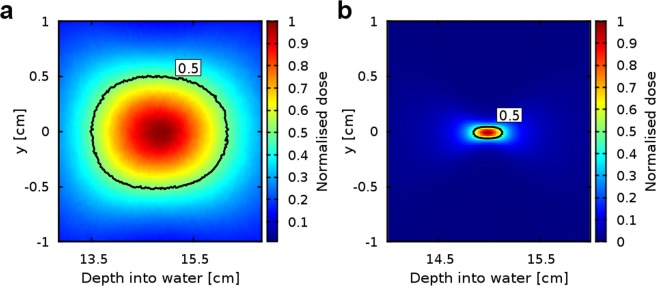
Samples of magnified dose profiles at the focus. (**a**) 200 MeV and (**b**) 2 GeV beams focused at a depth of 15 cm for *f/1.2*. The contour (black line) shows the iso-dose at full-width at half maximum. The dose is normalised to the maximum dose of a collimated beam.

The shape of the *volumetric element* was studied in detail for 200 MeV and 2 GeV beams and all *f-numbers* by performing simulations using a high-resolution scoring mesh in the dose peak region. Magnified lateral dose profiles (shown in Fig. 4 for *f/1.2*) were symmetrical with respect to the propagation axis, for both energies and all *f*-*numbers*. The transverse and longitudinal sizes were calculated by fitting Gaussian and Cauchy-Lorentz functions, respectively. The longitudinal positions of the dose peak, full-width-at-half-maximum in longitudinal (FWHM_z_) and transverse (FWHM_x_) directions are summarised in Table 1. FWHM_x_/FWHM_z_ ratios for the selected *f-numbers* were in the range 0.1–0.8 for 200 MeV and 0.08–1 for 2 GeV, respectively, where values close to 1 indicate near-spherical shape. Because of reduced scattering, 2 GeV beams produced the smallest and most symmetrical shapes, with radii down to 0.3 cm. For 200 MeV, the transverse size was 0.6–1.0 cm for the chosen *f-numbers*.

**Table 1:** Peak (D_max_) positions and FWHM of the *volumetric elements* for 200 MeV and 2 GeV electron beams focused at 15 cm from the entrance of the water phantom for *f/11.5*-*f/1.2*.

f-number	200 MeV			2 GeV		
	FWHM_z_ (cm)	FWHM_x_ (cm)	Peak position (cm)	FWHM_z_ (cm)	FWHM_x_ (cm)	Peak position (cm)
1.2	1.26	0.97	14.58	0.33	0.32	14.71
1.4	1.37	0.93	14.55	0.35	0.31	14.71
1.6	1.50	0.89	14.51	0.41	0.29	14.71
1.9	1.67	0.86	14.45	0.48	0.28	14.71
2.3	1.91	0.82	14.36	0.58	0.27	14.71
2.8	2.27	0.79	14.20	0.73	0.26	14.72
3.8	2.84	0.77	13.87	0.98	0.26	14.73
5.7	3.82	0.72	13.06	1.47	0.25	14.75
11.5	5.57	0.61	10.50	2.97	0.25	14.85

Total (D_tot_) doses integrated over the phantom volume are presented in Table 2 together with maximum and integrated surface (D_surf,max_, D_surf,int_) and exit (D_exit,max_, D_exit,int_) doses normalised to a 2 Gy peak dose (D_max_), modelling a typical radiotherapy fraction^27^. For strongly focused beams (*f/1.2*-*f/2.8*) and 200 MeV energy, D_surf,max_ was reduced by 40–211 times, and D_exit,max_ 20–54 times compared to *f*/∞. For 2 GeV energy D_surf,max_ decreased from 250 to 1800 times and D_exit,max_ from 230 to 1100 times than for *f*/∞. The total dose in the phantom decreased by 77.8–84.9% for 200 MeV and 97.2–97.5% for 2 GeV. Entrance and exit doses obtained for 2 GeV energies were smaller for all *f-numbers*, because the 2 Gy target dose can be delivered by a lower charge electron beam.

**Table 2:** Maximum, surface, exit and total doses for 200 MeV and 2 GeV electron beams focused at 15 cm from the entrance of the water phantom for *f/11.5* - *f/1.2*. The doses are normalised to a single fraction of 2 Gy delivered in the peak (D_max_).

f-number	200 MeV					2 GeV				
	D_tot_ (Gy)	D_surf,max_ (Gy)	D_surf,int_ (Gy)	D_exit,max_ (Gy)	D_exit, int_ (Gy)	D_tot_ (Gy)	D_surf,max_ (Gy)	D_surf,int_ (Gy)	D_exit,max_ (Gy)	D_exit,int_ (Gy)
1.2	108.55	0.01	0.38	0.02	0.49	9.53	0.00	0.02	0.00	0.07
1.4	99.78	0.01	0.36	0.01	0.47	9.18	0.00	0.02	0.00	0.07
1.6	92.07	0.01	0.34	0.02	0.45	8.89	0.00	0.02	0.00	0.07
1.9	85.02	0.02	0.31	0.03	0.43	8.65	0.00	0.02	0.00	0.07
2.3	79.18	0.03	0.29	0.03	0.40	8.47	0.00	0.02	0.01	0.07
2.8	73.68	0.04	0.27	0.04	0.38	8.34	0.00	0.02	0.01	0.06
3.8	68.59	0.07	0.25	0.06	0.35	8.25	0.01	0.02	0.02	0.06
5.7	61.35	0.14	0.22	0.09	0.32	8.18	0.01	0.02	0.03	0.06
11.5	44.46	0.40	0.16	0.11	0.23	8.15	0.05	0.02	0.11	0.06
∞	488.24	1.67	1.80	0.86	2.53	340.37	0.85	0.91	1.98	2.62

**Figure 5:**
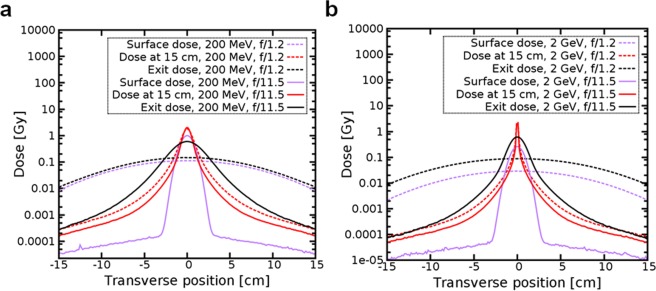
Surface dose, dose at 15 cm and exit dose as a function of the distance from the central axis. The electron beam is focussed at 15 cm from the entrance of the water phantom for *f/1.2* and *f/11.5* and has energy of (**a**) 200 MeV and (**b**) 2 GeV. The dose is normalised to a single fraction of 2 Gy in the peak.

Figure 5 shows cross-sections of lateral dose distributions at the surface, exit and 15 cm depth for *f/1.2* and *f/11.5* for 200 MeV (**a**) and 2 GeV energies (**b**). Surface and exit dose had similar profile and value for 200 MeV and *f/1.2*, whereas for 2 GeV the surface dose was 7 times lower than the exit dose. For *f/11.5* and 200 MeV the peak dose at the surface was almost as high as the peak dose, whereas for *f/1.2* it was more than 10 times lower. The peak dose at 15 cm for 2 GeV was two orders of magnitude higher than at the surface and more than one order of magnitude higher than at the exit.

**Figure 6:**
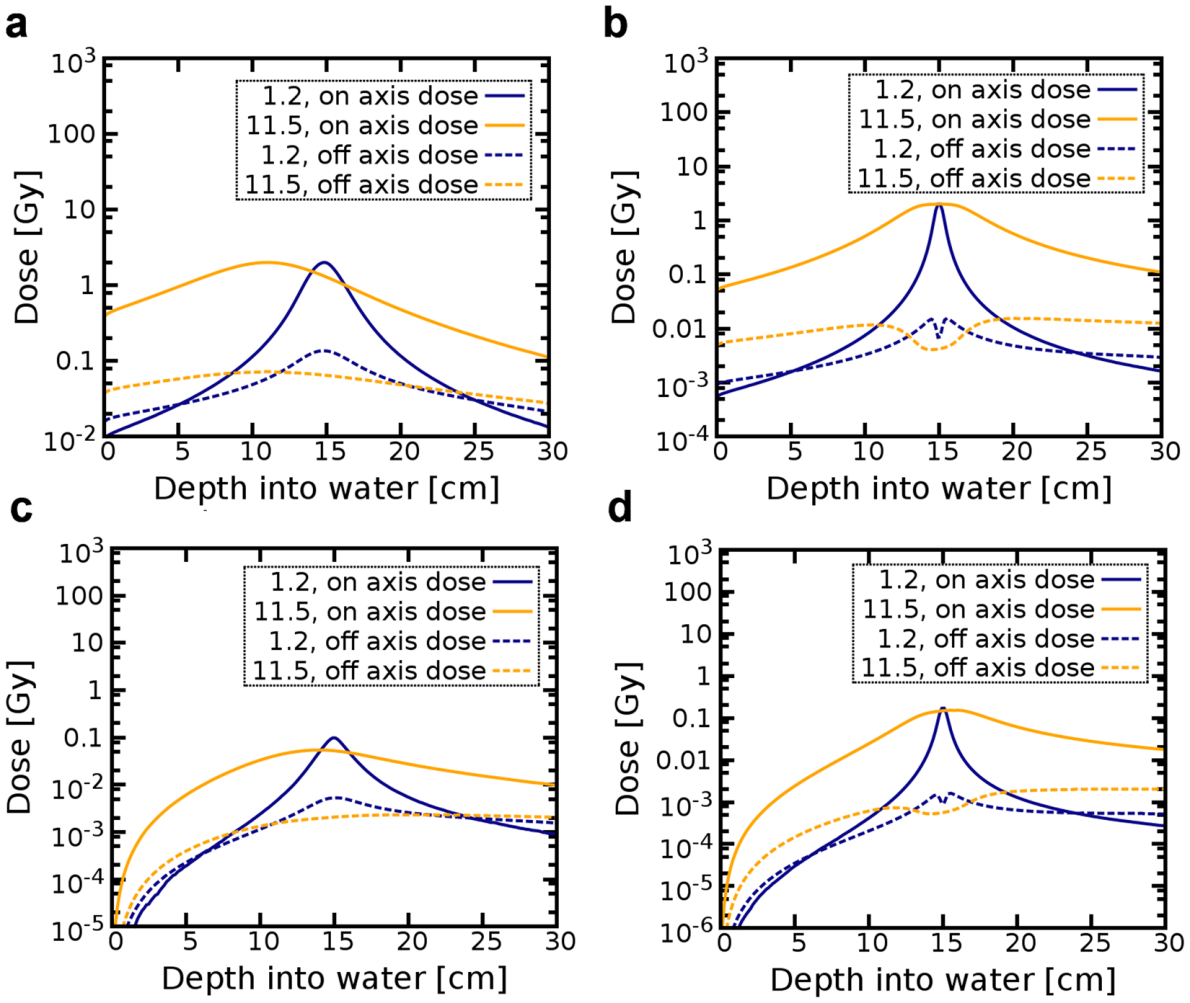
On-axis and off-axis dose as a function of depth for 200 MeV and 2 GeV electron beams focused at 15 cm from the entrance of the water phantom for *f/1.2* and *f/11.5*. Plots show (**a,b**) the total and (**c,d**) positron dose for 200 MeV and 2 GeV electron energies, respectively.

Figure 6 shows longitudinal total and position dose distributions integrated transversally on-axis and off-axis, as described in Methods, and normalised to a 2 Gy target dose. The off-axis dose is slightly higher than the on-axis dose at the entrance and exit of the phantom for both energies and *f/1.2*, while it is significantly higher across the phantom for *f/11.5*. A small drop is observed in the off-axis dose distribution for 2 GeV due to the small beam size at focus, which leads to dose deposition mostly on-axis.

Electrons traveling in water produce photons, initiating a cascade of interactions that leads to the generation of particles that have different relative biological effectiveness (RBE). Photons with energy above 1.02 MeV create electron-positron pairs. Neutrons can be produced by photons with 10–19 MeV energy in low-Z materials (up to Z = 20, such as H, C, N and O) and 4–6 MeV in high-Z (above Z = 20, such as Ca) materials. Photons with energies above 211 MeV produce muon pairs^28^.

Our simulations showed that for a focused electron beam the density distribution of secondary photons and positrons are also concentrated into the target volume. For all chosen *f-numbers* positrons were responsible for up to 4% and 11% of the total dose for 200 MeV and 2 GeV, respectively. Their depth-dose distribution was similar to the total dose, both on-axis and off-axis, except for a slower build-up at the phantom surface (Fig. 6). Muons were only produced for GeV beams and their contributions to the total dose was about 10^−8^ Gy for a 2 Gy target dose. Neutrons deposited about 10^−12^ Gy for both energies and 2 Gy target doses.

**Figure 7:**
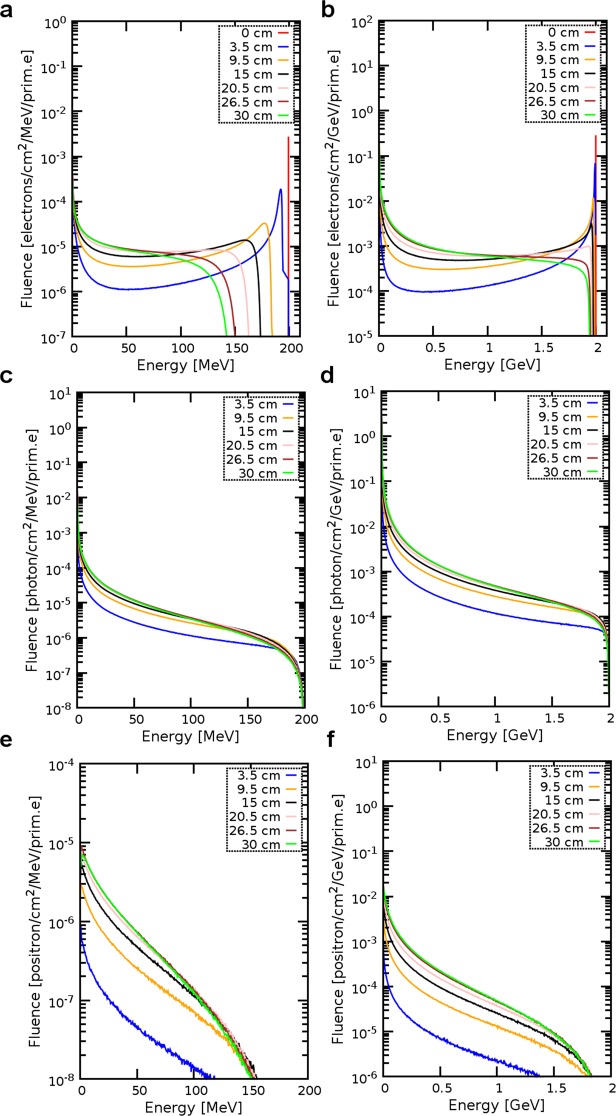
Energy spectra of electrons, photons and positrons at selected depths in the phantom and generated by an electron beam focused at 15 cm with *f/1.2*. Electron fluence for (**a**) 200 MeV and (**b**) 2 GeV initial electron energies. Photon fluence for (**c**) 200 MeV and (**d**) 2 GeV initial electron energies. Positron fluence for **(e)** 200 MeV and (**f**) 2 GeV initial electron energies.

Figure 7 shows the electron, positron and photon energy spectra at various depths in the water phantom generated by 200 MeV and 2 GeV electron beams for *f/1.2*. Electron beams were initially mono-energetic and travelling in vacuum, therefore energy spectra at the entrance of the phantom were single narrow peaks. As the beam propagated inside the water, electrons lost energy through collisions and emitted bremsstrahlung radiation, resulting in a rapid broadening of the spectrum and a shift to lower energies.

Photon spectra presented in Fig. 7c,d show that the photon fluence decreased monotonically with energy, forming a broad spectrum that extended to the maximum electron energy (bremsstrahlung endpoint). Positron spectra presented in Fig. 7e,f show their yield and distribution to be approximately constant at depths beyond 15 cm. Most positrons had low energies and annihilated with core electrons leading to the ejection of atomic electrons, and emission of characteristic X-rays and γ-ray photons.

We have considered the level of activation produced when focused VHEEs interact with dense materials. Simulations performed for layers of bone structure and skeletal muscle embedded in the water show that radioactive isotopes such as ^10^C, ^11^Be, ^14^N and ^23^Ne are formed. Assuming strong focusing (*f/1.2*) and delivery of 2 Gy of dose per target in one second, the total activity after one minute irradiation was about 240 Bq for 200 MeV, and 600 Bq for 2 GeV, which decreased to 5 Bq for 200 MeV and 9 Bq for 2 GeV after one hour. The maximum dose due to radioactive products was concentrated close to the focal spot, but was 5–6 orders of magnitude lower than the target dose (2 Gy) after 1 min. The dose in the bones and muscles from induced radioactivity decreased by a factor of 20 and 4 from 1 minute to 1 hour after irradiation for 200 MeV and 2 GeV beams, respectively.

## Discussion

Our study using FLUKA Monte Carlo simulations demonstrates that focused VHEEs can deliver a high dose to deep seated tumours, whilst reducing the dose absorbed by surrounding volumes. The simulations show that a small *volumetric element*, covering a volume of around 0.1–1 cm^3^, can be created at a chosen depth. Irregular shape and large size tumours can be treated by shaping the initial electron beam spatial and energy distribution, or by scanning the focused beam over the desired volume. However, further work is required to identify optimal delivery methods, ideally by integrating focused VHEEs into treatment planning systems.

The largest fraction of the dose is deposited by low-energy electrons and positrons, with approximately 27% of the total dose arising from particles produced by photon interactions. The contribution from neutrons and induced radioactivity to the total dose in the phantom is up to 0.003% for a 2 GeV beam and one order of magnitude lower for 200 MeV.

The majority of secondary particles in VHEE therapy, i.e. electrons, photons and positrons, are low linear energy transfer (LET) particles. The radiation damage produced by neutrons and induced radioactivity could be a concern because of the corresponding high biological response of tissue. They have high LET and can induce significant radiation damage even if present in small numbers. Our simulations show that about 10^−4^ and 10^−3^ neutrons/prim.e are produced in the energy range of 0.1–10 MeV, where the RBE is up to 20 times higher than for photons^29^, with 200 MeV beams and 2 GeV, respectively. Assuming a 2 Gy fraction delivered to the target, we estimate that about 10^5^ neutrons would be produced.

A challenge in cancer treatment is improving the precision of dose delivery. The tumour position is often not fixed during treatment and can move by as much as the tumour size during extended treatment times^30,31^. Therefore, use of a single beam, as considered here, would be of value when tumour motion is important.

An alternative solution for reducing the surface dose in VHEE therapy is High-Energy Agile Scanning Electron Radiotherapy (PHASER)^32^, which allows 360° treatment using a steerable VHEE beam. Current advances in laser-plasma wakefield accelerators^33^ create an opportunity for more compact, cost-effective and efficient methods of delivering VHEE therapy by dividing a laser beam into several beams using mirrors, where each mm long accelerator produces a VHEE beam in a treatment delivery room^34^.

## Conclusions

We have carried out Monte Carlo simulations to investigate how focused VHEE radiotherapy could be used for treatment of deep-seated tumours. Our study shows that VHEEs can be focused into tissue to create well-defined and precise concentrations of high dose in small volumes, while simultaneously reducing radiation dose to surrounding healthy tissue. Central-axis depth-dose profiles indicated lower surface and exit doses, compared with collimated VHEE beams.

VHEEs interacting with tissue are a source of mixed radiation, comprising mostly secondary electrons, photons, positrons and neutrons. Focusing provides a unique degree of control, with the possibility of using either a scanning mode or a single beam, delivered in a short time. Further work is required to design a beam transport system and develop treatment planning systems. Accelerator centres, such as CERN, have produced high-gradient X-band electron accelerators and strong magnetic quadrupoles with large bore sizes that could be practically implemented.

## Methods

### Monte Carlo simulations

The Monte Carlo code FLUKA (FLUktuierende KAskade)^21^ (version 2011.2c.6) is used to simulate the transport and interaction of charged particles and photons with a 30 × 30 × 30 cm^3^ water phantom (tissue equivalent material) surrounded by vacuum. The FLUKA code has been benchmarked against Geant4^35,36^, which has been shown to be a reliable tool for accurate calculations involving clinical data^37^. Details of the physics settings, beam parameters and detector configurations are given below. A simulation of a 200 MeV electron beam, using 100,000 primaries and the full physics for neutrons, takes about 35 min (average of 10 runs) on a workstation with an Intel Xeon CPU E5-2640 v3 @ 2.60 GHz. The execution time increases approximately linearly with the number of primaries. FLUKA does not support multiprocessing, as implemented for example in Geant4, but different cycles can be run in parallel.

### Data processing and visualisation

Post processing (merging) of the output data files is performed with FLUKA routines specific to each FLUKA-defined detector type depending on the scoring quantity. Two types of detectors were used in the simulations, USRBIN for dose calculation and USRBDX for fluence calculation. The 2D dose distribution in Fig. 3 is generated using Matlab (release 2017a).

### Percentage depth-dose distribution

The percentage depth doses (PDDs) are calculated for electron, photon and proton beams, as shown in Fig. 1. Simulations are performed using 10^7^ histories and 5 cycles, except when simulating a photon beam, for which the number of cycles is 15, in order to minimize the stochastic error arising from the random seed generator of the code. In all cases, the beam has a Gaussian transverse distribution with full-width-at-half-maximum (FWHM) of 15.9 cm at the phantom entrance. This value has been chosen to match the size of a focused electron beam at the water phantom with *f/1.2* and is large enough to produce a quasi-uniform dose distribution in the scoring volume, which consists of a USRBIN rectangular mesh with cross-sectional area of 3 × 3 mm^2^ and length of 30 cm located at the centre of the phantom. The resolution of the mesh is 1.5 mm in all directions.

The photon beam is collimated and its energy spectrum matches the output of a 6 MV Elekta Synergy LINAC.

Simulations for protons are performed enabling the HADRONTHERAPY setting and the EVAPORATion and COALESCEnce physics cards. A full heavy particle transport is activated. Two configurations are considered: in one case (Fig. 1, curve **b**) the proton beam is collimated and monoenergetic, with 147 MeV energy; the second case (Fig. 1, curve **c**) reproduces a Spread-out-Bragg peak (SOBP) using the depth-dose data obtained for a collimated proton beam with nominal energy of 160 MeV.

Electron beams are simulated for 200 MeV and 2 GeV energy, with mono-energetic distribution, using both collimated and focusing (*f/1.2*) geometry. The physics cards are specified in the next section.

### Focused VHEE beams

Simulations are performed for 10^7^ histories (approximately 1.7 pC charge) and 5 cycles, which resulted in statistical uncertainties of less than 5% in the peak region of the absorbed dose distributions, except for the simulations shown in Fig. 6, which use 10^7^ particles and 15 cycles to further improve the statistical error. The dose outside of the peak regions for all simulations is calculated with the uncertainty below 3–4%. We consider initially mono-energetic electron beams with energy of 200 MeV and 2 GeV. The initial transverse distribution is Gaussian with an r.m.s. radius of 10 cm. Electrons converge towards a point at the centre of the phantom, 15 cm from the entrance. The phantom position and the source size are fixed, while the source position is translated to match the *f-number*. A set of *f-numbers* between 1.2 and 11.5 is used, corresponding to distances between the source and the phantom of 9.1, 12.5, 16.7, 22.3, 30.1, 41.7, 61, 99.3, 214 cm. Simulations are performed using the PRECISIOn physics setting, which provides interaction models for all electromagnetic and nuclear processes that are relevant to the transport of VHEEs, including gamma interactions with nuclei. Photonuclear reactions such as delta resonance, quasi-deuteron and giant dipole resonance are enabled. The threshold for transport and production of delta particles and photons (ECUT, PCUT) is set to 10 keV in water with an EMCUT card. Survival probability of a single photon that is produced via hadronic interactions is set to 0.002 and 0.02 for secondary photons for 200 MeV and 2 GeV, respectively. The energy transfer to electrons lower than the threshold is estimated according to the continuous slowing down approximation. Production of photons, secondary electrons (based on Moller theory^38^) and positrons (based on Bhabha theory^39^) via interactions with atomic electrons is simulated explicitly above this threshold.

### Dose distribution

The dose distribution deposited in water is scored using a 30 × 30 × 30 cm^3^ USRBIN mesh that covers the whole phantom and has a resolution of 1.5 mm in all directions. Different USRBIN cards are used to score the total dose deposited by all particles, as well as the separate contributions from positrons. The integral surface and exit doses are calculated in a 0.15 cm thick slice of the phantom at the entrance and exit. The peak surface and exit doses are simulated within 0.15 × 30 × 0.15 cm^3^ volume at the entrance and exit of the phantom. The on-axis dose plotted in Fig. 6 is calculated in a longitudinal 0.3 × 0.3 × 30 cm^3^ slice at the centre of the phantom. The off-axis dose corresponds to the integrated dose deposited in the rest of the phantom, outside of this central slice. A smaller (4 × 4 × 4 cm^3^) high resolution (0.2 mm in the transverse and 0.1 mm in the longitudinal direction) mesh is used for the 2D transverse profile plots shown in Fig. 5.

### Energy spectra

The energy spectrum of electrons, photons and positrons is obtained using USRBDX cards to score the fluence of particles crossing the boundaries of thin 0.01 × 30 × 30 cm^3^ rectangular slices placed orthogonally to the propagation axis at depths of 0, 3.5, 9.5, 15, 20.5, 26.5 and 30 cm from the entrance of the water phantom. The USRBDX cards are set to score only outgoing particles, providing a linear binning in energy and solid angle, with 444 energy bins and one angular bin.

### Photon and neutron yield

Different USRBDX cards are used to calculate the yield of neutrons with energy <20 MeV (44 energy bins) and >20 MeV (400 energy bins). The photon and neutron yield was estimated with statistical uncertainty below 4%. The additional PHOTONUCLEAR card is activated to include electronuclear interactions at all electron energies and physics cards with options COALESCEnce and EVAPORATion are activated for all secondary particles.

### Neutrons

Processes specific to neutron production, such as coalescence and evaporation, are activated in the input file by relevant PHYSICS cards. The LOW-NEUTRON card is activated for neutron energy groups up to 260 (2 × 10^−14^–0.02 GeV) and gamma groups up to 42 (10^−6^–10^−5^ GeV) with a maximum energy of the low-energy cross section neutrons. The energy range for specific groups is defined in the FLUKA manual^21^. The dose is scored with a USRBIN detector filtered by an AUXSCORE card.

### Muons

The IONTRANS card is activated in order to turn on full transport of all light and heavy ions. Delta ray production is activated by the card DELTARAY with a kinetic energy threshold set to 100 keV. The default step size of muons is changed to 0.02 (fraction of the kinetic energy) using the FLUKAFIX cards. Particle transport threshold is set to 10 keV for muons and anti-muons with the card PART-THR. The additional PHOTONUClear card is activated by the option MUMUPAIR, with the interaction length biasing factor set to 10^−4^ to achieve better statistics of muon pair production by photons. The mechanism of muon interaction is controlled by the activated cards PAIRBREM, with the kinetic energy threshold for bremsstrahlung and pair production set to 10 keV and MUPHOTONs.

### Radionuclide production

Two layers of skeletal muscle and two layers of compact bone, all with 3 cm thickness, are inserted into the water phantom at 5, 8, 16 and 19 cm from the entrance, respectively. Materials are defined according to the ICRU Report 37, with a density of 1.04 g/cm^3^ and 1.85 g/cm^3^, respectively. The IRRPROFI card is used to define an irradiation profile that delivers 7.9 × 10^11^ and 0.46 × 10^11^ electrons in a 1 s (corresponding to 2 Gy) interval for 200 MeV and 2 GeV electron beam, respectively. The IONTRANS card is included to account for the full transport of all light and heavy ions. Hadronic interactions are modelled with the high-precision PEANUT model activated at all energies. The activity from the produced radioactive nuclides is calculated using RESNUCLEi cards with the DCYSCORE card set to 1 minute, 1 hour and 24 hours.

## Data Availability

Data associated with research published in this paper is available at 10.15129/aeaa36d6-8154-44bd-9575-af688c09f4b7.
